# RNAscope *in situ* hybridization confirms mRNA integrity in formalin-fixed, paraffin-embedded cancer tissue samples

**DOI:** 10.18632/oncotarget.21851

**Published:** 2017-10-16

**Authors:** Victoria Bingham, Leanne McIlreavey, Christine Greene, Edwina O’Doherty, Rebecca Clarke, Stephanie Craig, Manuel Salto-Tellez, Stephen McQuaid, Claire Lewis, Jacqueline James

**Affiliations:** ^1^ Northern Ireland Biobank, Centre for Cancer Research and Cell Biology, Queen’s University, Belfast, UK; ^2^ Molecular Pathology Programme, Centre for Cancer Research and Cell Biology, Queen’s University, Belfast, UK; ^3^ Tissue Pathology, Belfast Health and Social Care Trust, Belfast City Hospital, Belfast, UK

**Keywords:** mRNA, FFPE, *in situ* hybridization, Integrity, Pathology Section

## Abstract

Immunohistochemistry remains the overwhelming technique of choice for test biomarker evaluation in both clinical or research settings when using formalin-fixed, paraffin embedded tissue sections. However, validations can be complex with significant issues about specificity, sensitivity and reproducibility. The vast array of commercially available antibodies from many vendors may also lead to non-standard approaches which are difficult to cross-reference. In contrast mRNA detection, by in situ hybridization (ISH) with sequence specific probes, offers a realistic alternative, with less validation steps and more stringent and reproducible assessment criteria. In the present study mRNA ISH was evaluated in prospectively and retrospectively collected FFPE samples within a cancer biobank setting. Three positive control probes, POLR2A, PPIB and UBC were applied to FFPE sections from a range of tumour types in FFPE whole-face (prospective collection) or TMA (retrospective collection) formats and evaluated semi-quantitatively and by image analysis. Results indicate that mRNA can be robustly evaluated by ISH in prospectively and retrospectively collected tissue samples. Furthermore, for 2 important test biomarkers, PD-L1 and c-MET, we show that mRNA ISH is a technology that can be applied with confidence in the majority of tissue samples because there are quantifiable levels of control probes indicating overall mRNA integrity.

## INTRODUCTION

Detection of mRNA in formalin-fixed, paraffin embedded (FFPE) tissue samples by chromogenic RNA in situ technology has become a reliable alternative for a wide range of biomarkers in many areas of research including cancer and neurosciences [1-3]. However, the quality of FFPE samples retrieved from storage archives following routine pathology management pathways which may have relatively wide variations in fixation times compared to prospectively targeted biobank collections has not been fully established. Indeed there are contradictory opinions about the quality and quantity of nucleic acids that can be extracted from such FFPE collections and their potential use in downstream analysis [4].

RNAscope^®^ Technology provided by, Advanced Cell Diagnostics (ACD) is a very sensitive *in situ* hybridization technology. Based on ACD’s unique patented probe design strategy which enables simultaneous signal amplification and background noise suppression, RNAscope technology represents one of the most significant advances in ISH technology in over 40 years and there is clear evidence of the promise of RNAscope technology to address many of the biological or pathological challenges currently faced by scientists, such as biomarker interpretation in tissue, quantitation and heterogeneity of expression [5-7].

In general, immunohistochemical (IHC) assays may be inefficient with a lack of high quality antibodies for many newly discovered biomarker targets, coupled to extensive validation times and inconsistent performance. RNAscope is therefore highly attractive but must be suitable for detection of mRNA in multiple FFPE tissue samples with simple validation protocols. Furthermore, the technology should be robust enough to take account of acceptable variations in fixation parameters (time before fixation, time of fixation, time before dissection of gross resection specimen to optimal block size) which may present in large numbers of routine samples within a study cohort. This was demonstrated recently by a review of the challenges and pitfalls in detecting *PD-L1 expression in lung cancer*, by IHC [6]. Comparison of PD-L1 expression in different NSCLC clinical trials showed a high variation in PD-L1 prevalence. This variation can potentially be explained by differences in patient demographic, therapies and by variation in quality of assay antibodies or IHC methods used for detection. Alternative methods like RNAscope^®^ ISH have been tested to complement IHC analysis and it has been suggested that “mRNA ISH may identify patients that would benefit from immunotherapy that would otherwise be negative for PD-L1 protein expression by IHC” [6].

The purpose of the present study was twofold: Firstly, to assess, using 3 RNAscope control probes (POL2RA, PPIB and UBC), the suitability of RNAscope in four tumour types (colorectal, breast, prostate and ovarian) whereby FFPE tissue had been prospectively collected from patients consented to the Northern Ireland Biobank (NIB); secondly to assess the mid-range control probe PPIB expression and correlate to test probe biomarkers (PD-L1 and c-MET) in a series of 353 colorectal cancer (CRC) FFPE samples collected over a 5-year period within routine pathology practice in the Belfast Health and Social Care Trust (BHSCT). These samples were available within tissue microarray (TMA) format.

## RESULTS

### Prospectively collected FFPE samples – assessment of whole-face sections

**Figure 1:**
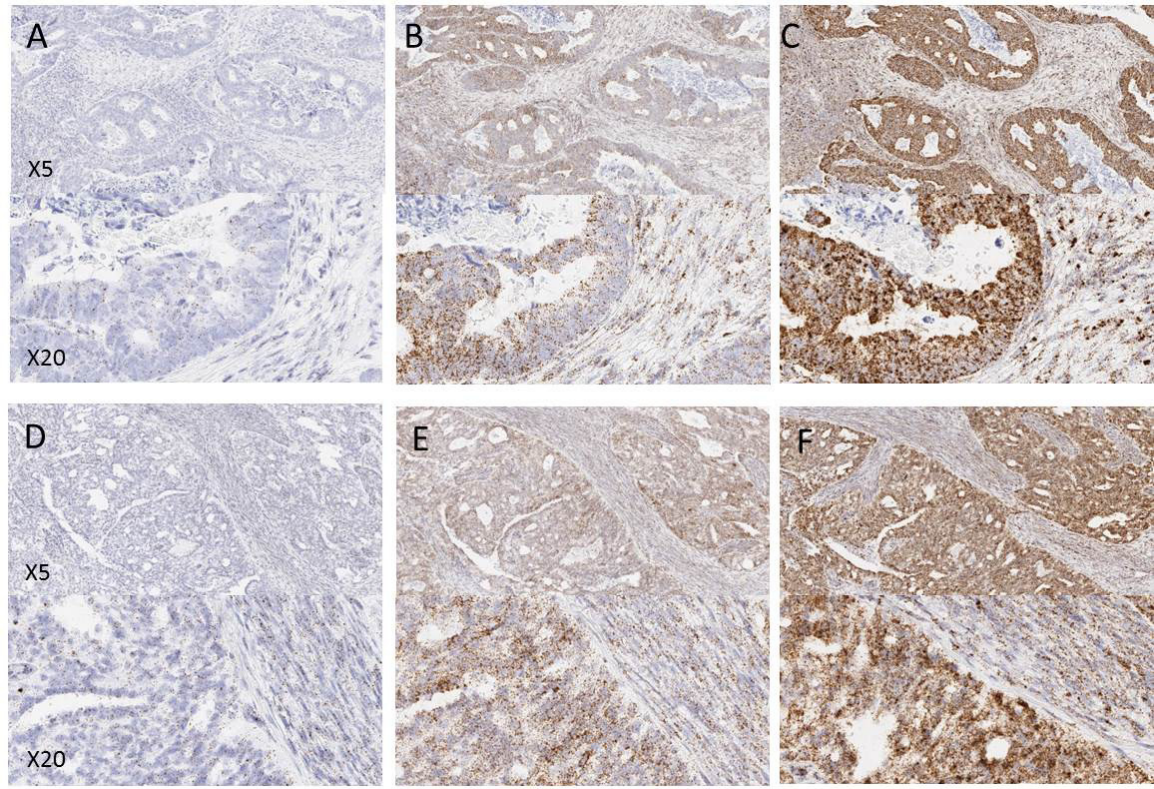
Examples of control probe expression in FFPE tissues from a prospectively collected cohort of samples (**A** and **D**) POLR2A, (**B** and **E**) PPIB, (**C** and **F**) UBC. A, B and C are examples from colorectal tissue, D, E and F are examples from ovarian tissue. Note the increasing expression level from POLR2A to UBC.

On initial microscopic evaluation the 4 tumour types showed uniform expression levels of the RNAscope control probes POLR2A, PPIB and UBC across the entire surface area of all tissue sections. Examples of expression in the tumour types are shown in Figure 1. In general probe expression was stronger in tumour epithelial cells than in surrounding regions of stroma (lymphoid or fibroblast cells). No expression of the negative control probe bacterial DapB was observed in any of the tissue samples.

**Figure 2:**
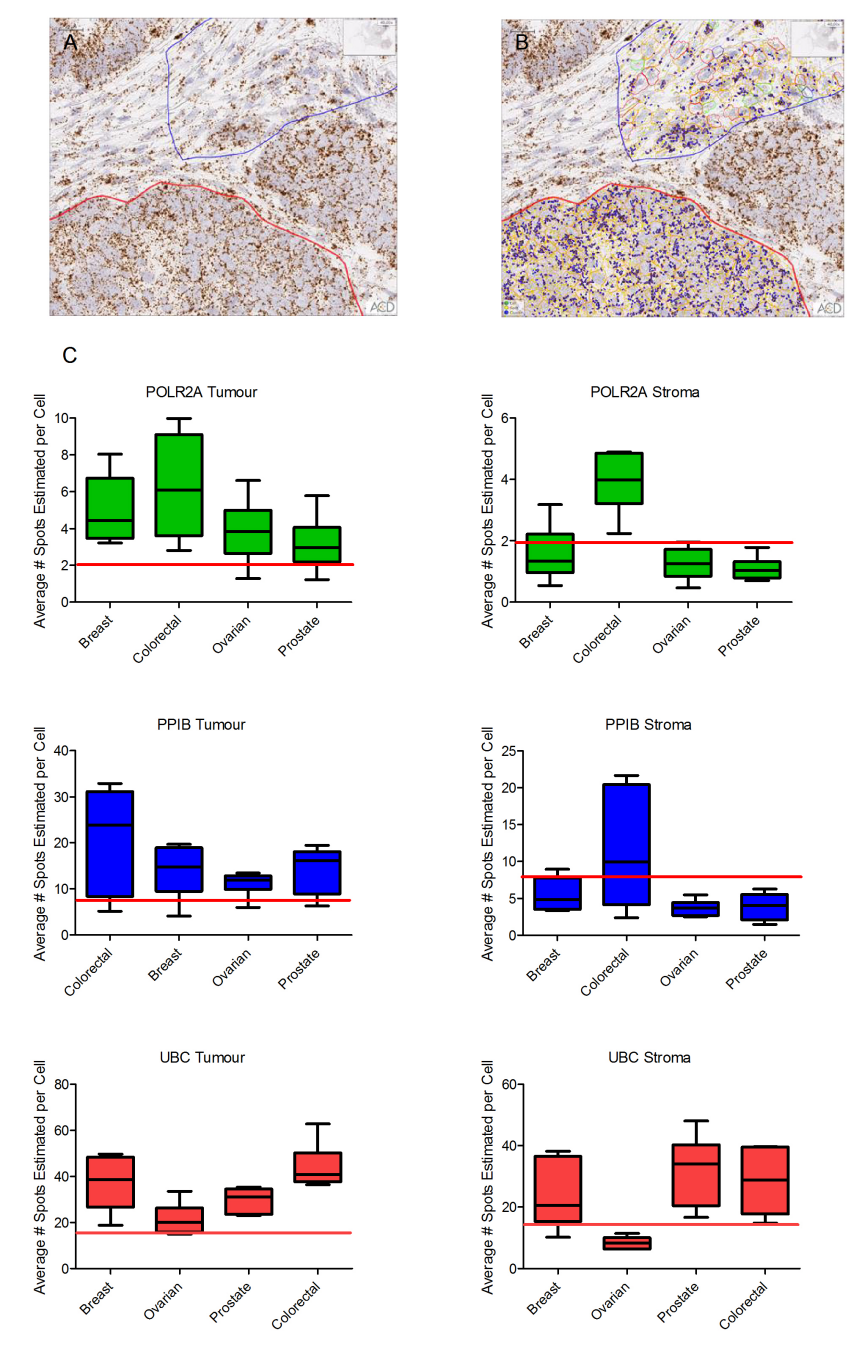
Quantitative expression by Spotstudio image analysis of three control probes in 6 cancer samples from 4 different tumour types (**A**). Region of tumour and stroma in a colorectal cancer case showing high expression of PPIB. (**B**). Example of the ROI selections for spot studio analysis in both tumour (red) and stroma (blue) compartments. Note the increased expression of PPIB in the tumour cells compared to stroma. (**C**) Graphed results are presented as average # spots estimated per cell in both tumour and stromal compartments. In all cases the horizontal red line depicts the lower threshold of expected expression. In almost all tumour compartments the expression of control probes would determine the tissues as fit-for-purpose for test biomarker analysis. Cut offs in stromal compartments are less robust.

Quantification of POLR2A, PPIB and UBC was performed using Spotstudio software in 6 distinct regions of interest (ROI), 3 from tumour and 3 from stromal compartment of each tissue block (see Figure 2A & 2B). Results are expressed as average number of spots per cell and are displayed as a box-and-whisker plot (min, max) with medians indicated by the horizontal bar (Figure 2C). This analysis confirmed the quality of the tumour tissue samples for mRNA analyses by In Situ Hybridization. For POLR2A, the lowest expressing control probe, all but two tumour regions (1 ovarian, 1 prostate) had at least 2 spots per cell and in many ROIs were much higher.

Similarly, for PPIB and UBC control probes, most tumour regions had an average number of spots per cell well within expected guidelines. The average number of spots per cell for PPIB was >8 in all but 1 case from each cohort. In all tumour compartments UBC probe expression was >15 spots-per-cell.

Expression of all three control probes was lower in cells in regions of stromal tissue compared to tumour regions (Figure 2C). For POLR2A most cells had at least 1 spot-per-cell. The range of PPIB expression in stroma was quite variable but in most cases was above 3 spots per-cell. UBC expression was generally high in stromal regions of tumour with a low of 6.29 in a single ovarian cancer case.

**Figure 3:**
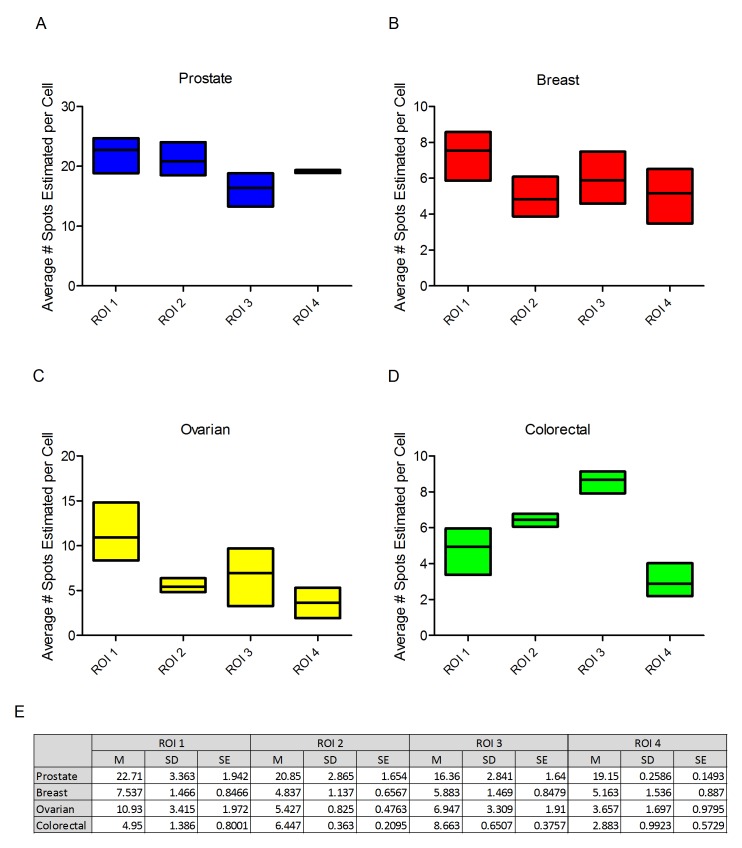
Boxplots depicting the average number of spots per cell of PPIB at three levels in four regions of interest (ROI) Prostate (**A**), breast (**B**), ovarian (**C**) and colorectal cancer (**D**). While variations are observed all ROIs irrespective of depth into the block would be suitable for test probe analysis. Table of descriptive statistics (**E**) summarising the mean, standard deviation and standard error between each level for the four ROIs.

From each tumour type a single example was selected for analysis of PPIB expression through the depth of the block at intervals of 100 microns. Four ROIs in the tumour compartment of each block were analysed at each of the 3 levels. Differences in expression levels were seen between the different ROIs across the tumour but reasonably consistent expression of PPIB was present at each of the three levels through the block (Figure 3). The most marked differences was observed in ROI 3 in ovarian tissue but the level with lowest expression would still be seen as fit-for-purpose for test probe analyses. The relatively low SDs and SEs suggest there is minimal variation between levels within single ROIs irrespective of depth in the block (Figure 3E).

### Colorectal cancer tissue microarray

**Figure 4:**
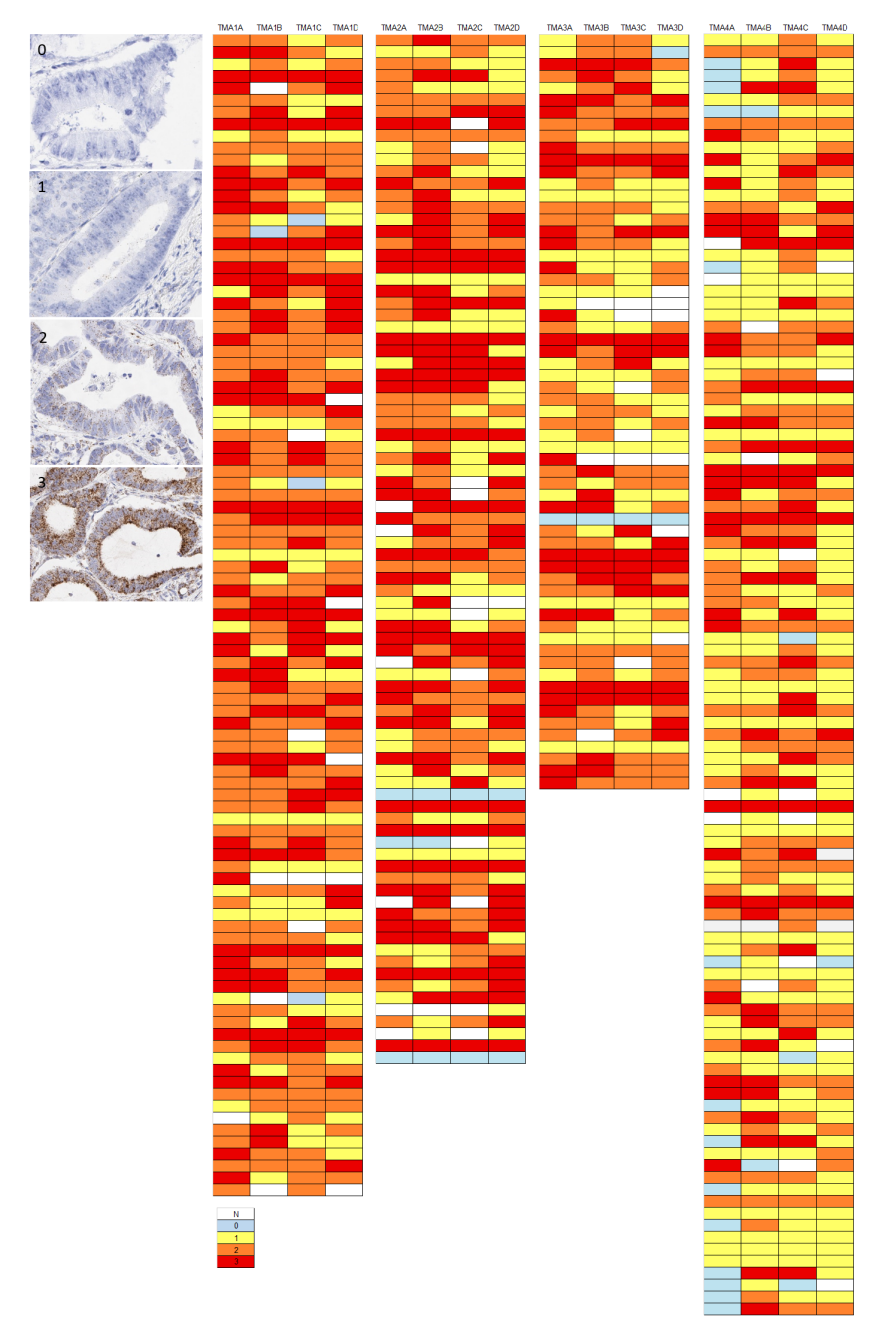
Examples of variation in PPIB expression (0-3) in individual TMA cores in a CRC series The accompanying illustration depicts the range of expression in 4 TMAs. Each TMA is represented by 4 replicate cores from the same donor FFPE block – 1A, 1B, 1C & 1D.

TMA sections were evaluated by RNAscope ISH for expression of the medium range control probe PPIB. Semi-quantitative microscopic analysis was performed by 3 individuals competent in RNAscope analyses (VB, SMcQ & LMcI). Results are summarised as a heat map for the 353 cases represented in the 4 TMAs analysed (Figure 4). The vast majority of cores across the various TMAs had either moderate or high numbers of spots in tumour cells indicating they had good integrity for test probe assessment. In only 2% of cores was absence of expression noted and in only 3/353 cases assessed (0.9%) was there absence of expression from all 4 cores derived from the donor tissue block.

**Figure 5:**
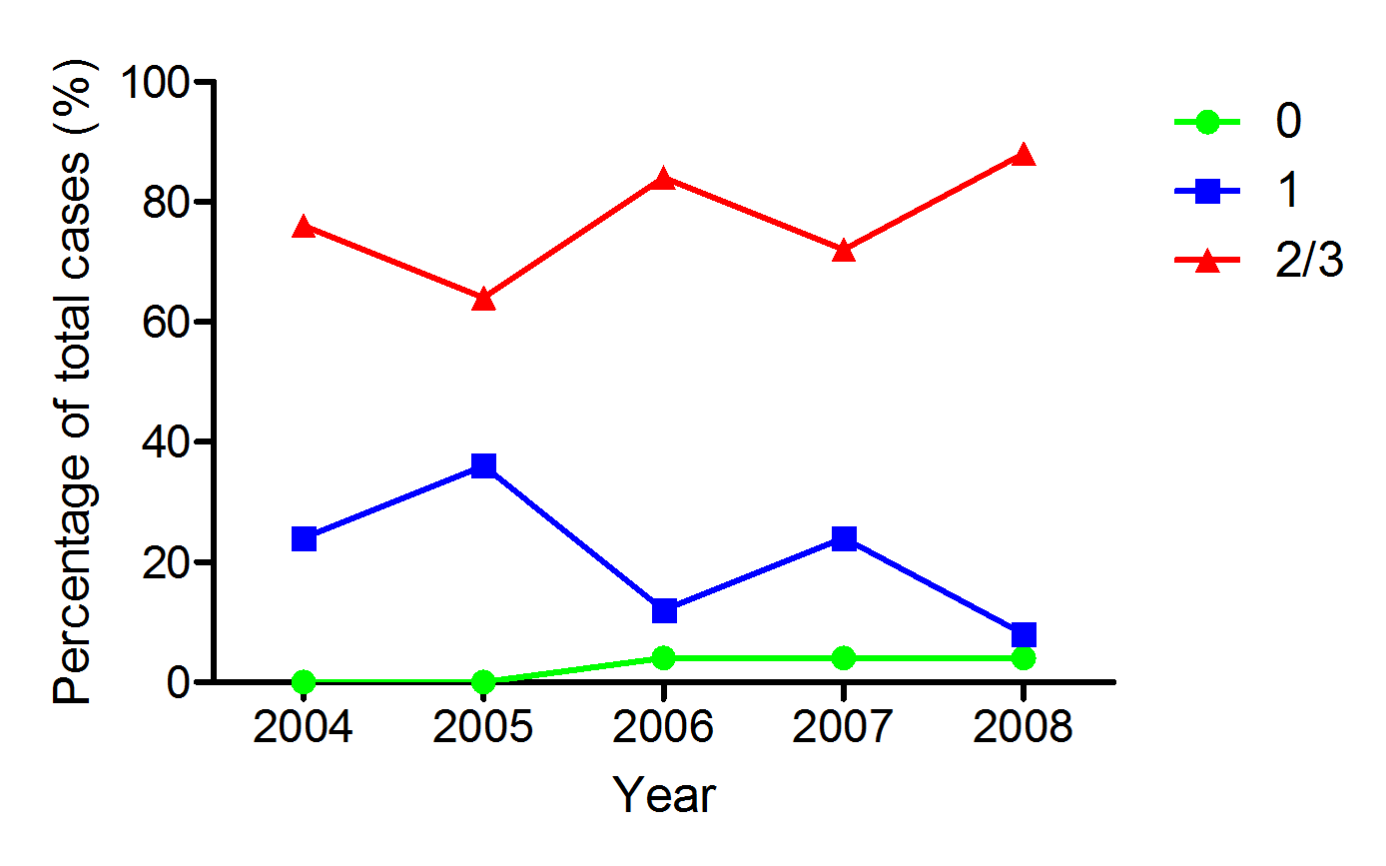
PPIB expression in TMA cores in a CRC series by block age of the donor FFPE sample Almost as many cases have moderate or high levels of expression in 2004 as in 2008. The rate of negative cores is actually higher in 2008 but still very low as a percentage of total cases. Green – PPIB score of 0; Blue – PPIB score of 1; Red – PPIB score of 2/3.

Analysis of PPIB expression by FFPE donor block age in years where at least 50 cases were available (2004-2008) demonstrated that there was no drop off in intensity of expression (Figure 5). Cases were as likely to have moderate or high levels of PPIB expression when the donor block was selected from 2004 as from 2008.

### Correlation of PPIB expression to PD-L1 and c-MET

**Figure 6:**
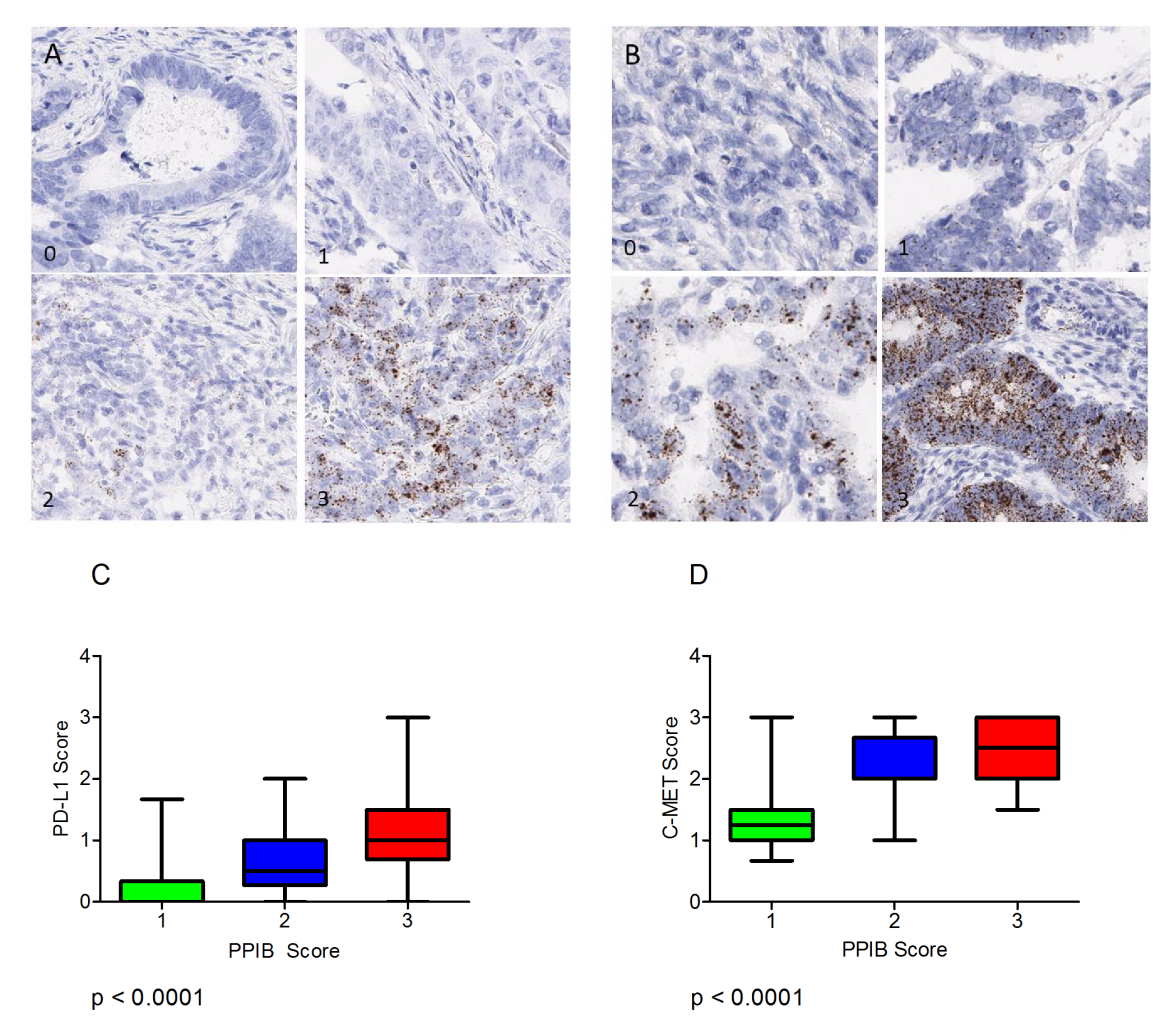
(**A**) Varying levels of PD-L1 expression (0-3) in CRC TMA cores. (**B**) Varying levels of MET expression (0-3) the CRC TMA cores. Boxplots depicting the manual scores for PD-L1 (**C**) and C-MET (**D**) compared to manual PPIB scores on TMA cores from 40 cases. A Kruskal-Wallis Test was performed to assess differences in test probe mean score versus cases with PPIB categorical scores 1-3. A p value of less than 0.05 was considered statistically significant.

Expression of the immune checkpoint biomarker PD-L1 and the receptor tyrosine kinase c-MET probes were also assessed microscopically in serial sections of the CRC TMAs and correlated to the level of expression of PPIB. For this analysis 120 cores were selected (40 PPIB score 1, 40 PPIB score 2 and 40 PPIB score 3). Expression variation is observed for PDL1 (Figure 6A) and C-MET (Figure 6B).

A Kruskal-Wallis test was performed to assess differences in PD-L1 and c-MET mean score versus cases with PPIB categorical scores 1-3 (Figure 6C & 6D). A significant difference was observed between both test probes and PPIB scores from 40 cases (p<0.0001). Data analysis would suggest that PD-L1 scores of 1, 2 or 3 are not affected by the expression of the control PPIB probe. However, in cores where no PD-L1 expression is observed then caution must be observed when then PPIB score is low. Similarly, c-MET RNAscope expressions of >1.5 do not appear to be affected by any level of PPIB expression.

**Figure 7:**
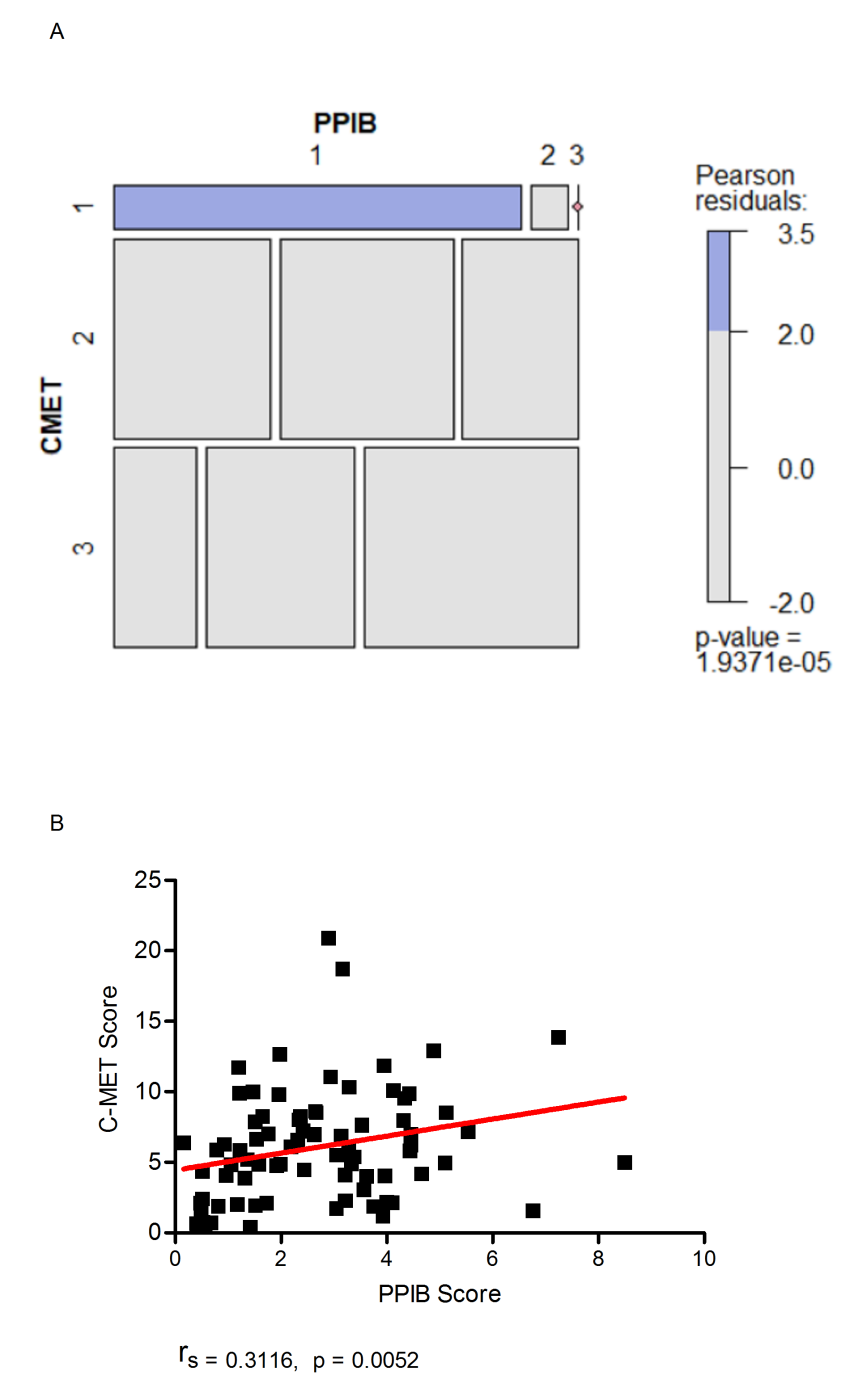
Mosaic plot (**A**) demonstrating individual contributions of categorical PPIB and c-MET scores with Pearson residual base shading. A p value of less than 0.05 was considered significant. Correlation plot (**B**) demonstrating the relationship of digital scores generated for c-MET to PPIB when a manual PPIB score of 1 was given. Correlation was calculated using Spearman’s Rank.

A Chi-Squared test of independence was performed to examine the relationship between qualitative spot studio image analysis scores generated for c-MET and PPIB. A significant interaction was found (X^2^ (4) = 27.056, p = 1.937e^-05^). Based on Pearson residuals of the test statistic, a greater than expected association in the number of cases with a c-MET and PPIB score of one was observed (Figure 7A) (Pearson residuals = 3.5, p < 0.05). Cases with a manual PPIB score of 1 were assessed further using SpotStudio image analysis for PPIB and c-MET expression (Figure 7B). A moderate correlation was observed between the 2 probes using Spearman’s Rank. (r_s_ = 0.3116, p = 0.0052). This data would indicate that in TMA cores with low c-MET scores then caution is required in assessment when the PPIB score is also low.

## DISCUSSION

The goal of this study was to determine whether the suitability of FFPE blocks for mRNA detection could be confirmed using RNAscope is situ hybridization technology. The analyses performed with 3 RNAscope control probes indicate that FFPE tissue samples, collected from different tumour types in a prospective biobank setting, are fit-for-purpose for in situ hybridization analysis with test biomarkers with expression levels either comparable with or above that expected from ACD recommendations. Analysis of the control probe PPIB at different levels of the FFPE blocks demonstrated that there was little variation in expression which suggests uniform fixation through at least 200 microns of tissue.

Furthermore we demonstrate that many hundreds of colorectal cancer patient FFPE samples acquired from a tissue pathology archive and placed in TMA format are robust for RNA analysis by RNAscope. These findings were most readily confirmed in the malignant epithelial cell compartment of the tumours. Correlation of donor FFPE block age to PPIB expression in the TMA series demonstrated that the RNAscope assay is robust in respect of FFPE block storage time. This is a very important finding for the ever increasing use of TMAs, derived from donor FFPE blocks from diagnostic or biobank archives, in biomarker discovery which because they need to be linked to long-term clinical follow up data may be stored for long periods of time before TMA construction and usage.

The three control probes used in the study have distinct characteristics most of which have been evaluated by quantitative gene expression technologies [9-10]. POLR2A, which encodes the largest subunit of RNA polymerase II, is a low copy, rigorous positive control which may be necessary for very low expressing targets. It can be an alternative to PPIB for proliferating tissues, like tumours, and also for some non-tumour tissues. PPIB, which encodes for a cyclosporine-binding protein, is the recommended positive control for most tissues whereby it is expressed at a sufficiently low level so as to provide a rigorous control for sample quality and technical performance. PPIB has been shown to be only moderately up-regulated in activated lymphocytes after 96 hours of gamma-radiation stimulation [11]. Other studies have also demonstrated the use of the PPIB probe as a reliable and consistent control with little or no background in multiple tissue samples [12]. UBC, which encodes for a polyubiquitin precursor protein, is a relatively high expressing control probe which may not be useful as a control for low expressing targets as it can lead to false negative results. Similarly, in the context of qRT-PCR the question of the correct endogenous control reference genes for normalization of data has been the subject of much discussion with several studies showing differences in the stability of references genes in a variety of tissues [13-16].

While immunohistochemistry is the most widely available tissue hybridization technique, it may not be suitable in a variety of technical, biological and diagnostic frameworks and antibodies require very robust validations to show specificity, selectivity and reproducibility [17-18]. Indeed the Rimm group have published quite complex algorithms for validation of antibodies involving selection of best available from vendors followed by testing in cell lines, whole-face sections and TMAs. Furthermore, via the International Working group for Antibody validations (IWGAV), they have proposed a set of guidelines for validating antibodies with five conceptual pillars one of which must be met as a minimum criterion to claim that a particular antibody has been adequately validated for a specific application [19]. While some published work has attempted to address issues of loss of tissue degradation and the effect of increasing cold ischemic times on expression levels of protein epitopes via a Tissue Quality Index (TQI) this has not progressed beyond proof-of-concept. Also the concept of TQI is still problematic in terms of pre-analytical variables (cold ischaemia, hypoxia, penetrative fixation), for choice of epitopes for certain biomarkers (phosphorylated proteins and post-translational modifications) and for different tissue types [20-21].

Chromogenic RNA hybridization testing, with sequence specific probes, may be a robust, specific technology which allows for easier validation and standardization and is more readily quantified. In situ analysis of RNA in single cells at single molecule sensitivity in clinical specimens is a valuable tool in the era of personalised medicine. There are less validation steps in the process and reduced reliance on other strategies to prove specificity. It is also important to emphasise that due to the almost total absence of background or non-specific staining, RNA ISH with sequence specific probes is less prone to inter-observer variation. Indeed automated quantitative RNA in situ hybridization by RNAscope has been used to resolve equivocal and/or heterogeneous HER2 status in invasive breast carcinoma [22]. In many instances such as commercial unavailability of antibodies, low abundance, extracellular targets or where the target cannot be detected by antibody e.g. secreted proteins then mRNA hybridization may be the assay of choice.

Control probe expression in the stromal cell populations was much more heterogeneous than in the tumour epithelial compartment which indicates that much more rigorous and cautious interpretation of test biomarkers in these tissue regions is required. This lower expression of control probes in stromal tissues may be explained in part because, compared to transcriptionally active large tumour cells, many stromal cells are resting and express very low levels of mRNA [23]. Furthermore it is undoubtedly more difficult to assess probe expression in stromal cell populations due to the large diversity of cell types.

In the context of single cell profiling to uncover events such as transcriptional signalling of c-MET associated with early invasive cancer events [7] or with potential in the clinical setting of invasive breast cancer to detect HER2 [22], the robustness and usefulness of a technology such as RNAscope may be hugely beneficial but only if underpinned by the use of controls such as those described in the present study.

While use of RNAscope to detect mRNAs is becoming very popular it should be remembered that there is a poor correlation generally reported between levels of mRNA and protein. The varied post-translational mechanisms involved in turning mRNA in to protein are not as yet sufficiently well-defined to calculate protein expression levels from mRNA. Also proteins differ significantly in their half-life and there may also be technical differences in protein vs mRNA detection methodologies that limit the ability to correlate between protein and mRNA [24].

Both test biomarkers used in this study are known to be differentially expressed in the tumour compartment of different colorectal cancer cases ranging from absence of expression through to high level of expression on tumour epithelial cells for PD-L1 [25], or from low to high numbers of expressing cells for c-MET [26-27]. The present study confirms that the detection of both these biomarkers, by RNA in situ hybridization, is robust across a large number of samples acquired from a tissue pathology archive. Indeed the potential use of sequence specific RNA probes to detect PD-L1 expression in the clinical tissue pathology setting may be attractive as at present there are four PD-L1 assays registered with the US Food and Drug Administration which use 4 separate PD-L1 antibodies on 2 different automated platforms and have different scoring systems [28]. For c-MET analysis in CRC there has been discrepant published data which may reflect not only differences in technical detection methods used (IHC, qRT-PCR, FISH) but also the well-recognised problem of the variety of subjective scoring criteria that are in use for IHC [29-34]. Is this context c-MET RNAscope may offer a realistic alternative for measuring c-MET expression in invasive CRC.

In summary our recommendation is firstly do not evaluate cases for test biomarker analysis where control probe expression is absent. Secondly, in cases where control probe expressions are marginally positive then establishment of a cut-off value is required which may vary between test biomarkers. Careful analysis of test biomarker scores are required to ensure the range of differential expression is not intrinsically linked to overall mRNA integrity.

## MATERIALS AND METHODS

### Processing of prospectively collected FFPE samples

Prospectively collected colorectal, breast, prostate and ovarian tissue FFPE samples were acquired from the Northern Ireland Biobank who have ethical approval for the collection of surplus tumour and non-tumour control tissue (fresh and FFPE) and matched blood samples from consented patients within the BHSCT (REC reference 16/NI/0030). All prospectively collected NIB FFPE samples were dissected in a UKAS accredited laboratory, processed on Tissue-Tek 6 Vacuum Infiltration Processors and embedded in paraffin wax creating FFPE tissue blocks. Thin (3µm) tissue sections were cut and stained with Haematoxylin and Eosin (H&E) on a Tissue-Tek Prisma staining machine. For the present study tissue samples with greater than 50% tumour content (proportion of the whole tissue that is occupied by tumour tissue) were selected for RNAscope.

### CRC tissue microarray construction

353 cases of CRC were identified from the FFPE tissue pathology archives of the BHSCT. The primary collection period of these samples was 2004-2009 with a mean storage period of 10 years. Under ethical approval from the Northern Ireland Biobank, representative tumour blocks were retrieved, and a new section was cut for H&E staining for annotation prior to TMA constructions. For each selected donor block, four representative areas were annotated for targeted coring (3 tumour, 1 stromal/invasive edge). TMAs were constructed using a manual tissue arrayer (Beecher Instruments, Silver Spring, MD, USA) as described previously [8]. The manual arrayer was used to extract 1-mm-diameter tissue cores from donor blocks for insertion into recipient blocks.

### RNAScope *in-situ* hybridization and image analysis

Manual chromogenic RNAScope was performed on sections from whole-face and TMA tissue blocks using company protocols. Briefly, sections were cut at 4µm, air dried overnight, baked at 60C for 1hour, dewaxed and air-dried before pre-treatments. For all tissue sections a standard pre-treatment protocol was used. Three RNAScope positive control probes from Advanced Cell Diagnostics (ACD) (3960 Point Eden Way Hayward, CA 94545) were used in this study: positive control probe Homo sapiens ubiquitin C (Hs-UBC) (310041 Accession # NM_021009); positive control probe Hs-PPIB (313910 Accession # NM_000942.4); positive control probe HS-POLR2A (310451 Accession NM_000937.4) and 1 negative control probe to a partial cds; dihydrodipicolinate reductase (bacterial DapB: 310043 Accession # EF191515). Test probes to the immune pathway associated biomarker PDL1 – Hs-CD274 (600861 Accession # NM_014143.3 – sequence region 124 - 1122) and the receptor tyrosine kinase c-MET Hs-MET (423101 Accession #NM_000245.2 – sequence region 175-6505) were also used to stain the CRC TMAs. Detection of specific probe binding sites was with RNAScope 2.5 HD Reagent kit – brown from ACD (Cat. No. 322300).

The numbers and types of tissue samples and method of assessment are summarised in Table 1. For semi-quantitative microscopical evaluations of control or test probe mRNA detection by RNAScope a 4-tier scoring system was developed: 0 – negative; 1 = few spots in most cells; 2 = moderate number of spots in all cells; 3 = high number of spots in all cells

**Table 1:** Summary of the tissues, probes and scoring methodology used.

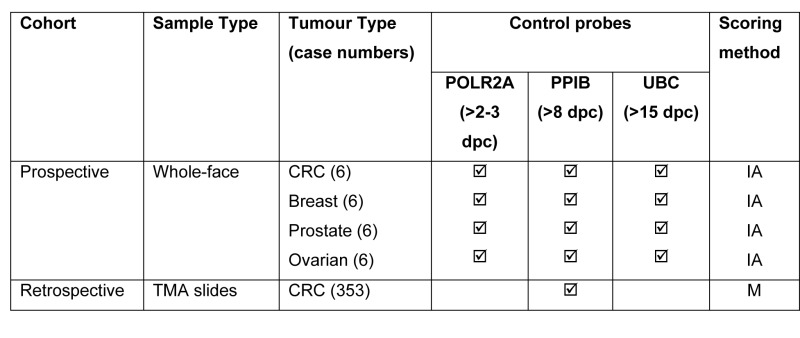

Key; Dots per Cell (dpc), Spotstudio Image Analysis (IA), Microscopic Evaluation (M)

Image analysis on selected regions of interest (ROIs) within the tumour or stromal compartments of control probe labelled whole-face sections from prospectively collected tissue samples was performed using Spotstudio™ Software from ACD with user-defined thresholds after slides were scanned using an Aperio scanner at x40 resolution. Sections were also cut and RNAscope performed for PPIB at three different levels, separated by 100 microns, through representative examples from each of the tumour types. Four comparable ROIs at each level were then subject to Spotstudio image analysis. Image analysis was also performed to assess c-MET mRNA expression against PPIB expression on a selected subset of CRC TMA cores.
